# Lactate–right ventricular discordance patterns and mortality in critical illness

**DOI:** 10.1186/s12872-026-06440-1

**Published:** 2026-08-12

**Authors:** Linzi Jin, Jun Xin, Lisi Ye, Zhenxia Wang, Tao Chen, Qinqin Shu, Yan Li

**Affiliations:** 1 Department of Emergency Medicine, Shanghai Fourth People’s Hospital, School of Medicine, Tongji University, Shanghai, 200434 China; 2 Department of Ultrasound, Shanghai Fourth People’s Hospital, School of Medicine, Tongji University, Shanghai, 200434 China

**Keywords:** Echocardiography, Lactate, Right ventricular dysfunction, Hemodynamic phenotyping, Critical care, MIMIC-IV, Supportive external assessment

## Abstract

**Background:**

Serum lactate and echocardiographic assessment are both central to hemodynamic evaluation in the ICU, yet their combined phenotypic information remains unexplored. We hypothesized that cross-classifying patients by lactate level and right ventricular (RV) function identifies clinically coherent classification groups with differential outcomes.

**Methods:**

Retrospective cohort study using MIMIC-IV v2.2 linked with MIMIC-IV-ECHO v1.0, with supportive external assessment in the eICU Collaborative Research Database. Adult ICU patients with transthoracic echocardiography and lactate measured within ± 6 h were classified by lactate (< 2 vs. ≥ 2 mmol/L) and qualitative RV function. Logistic models used patient-clustered robust standard errors; a sensitivity analysis retained only the earliest eligible observation per patient. Incremental value was assessed by adding RV dysfunction to a clinical model containing continuous lactate.

**Results:**

Among 6,228 observations from 4,965 patients (median age 66 years; 57.8% male; 30.9% hospital mortality), mortality increased from 18.8% in Low-Lactate/Preserved-RV to 47.2% in High-Lactate/RV-Dysfunction. Associations persisted with patient-clustered inference, first-day SOFA adjustment, and restriction to one observation per patient. Adding RV dysfunction to a clinical model containing continuous lactate produced a small improvement in AUC (0.758 to 0.761; ΔAUC 0.003, patient-bootstrap 95% CI 0.001–0.006). RR-based RERI was 0.070 (95% CI − 0.177 to 0.284). In 43,466 eICU stays with observed hospital outcome, diagnosis-coded RV disease reproduced the mortality ordering; aligned adjusted ORs were 2.01, 3.01, and 5.87 across the non-reference groups.

**Conclusions:**

Lactate–RV cross-classification identifies clinically coherent groups with graded mortality in ICU patients selected for echocardiography. RV assessment adds statistically detectable but modest prognostic information beyond continuous lactate and clinical covariates. The eICU findings support the prognostic pattern but do not constitute direct validation of the echocardiographic phenotype.

**Supplementary Information:**

The online version contains supplementary material available at https://doi.org/10.1186/s12872-026-06440-1.

## Introduction

Hemodynamic assessment in the intensive care unit relies on the integration of biochemical markers with imaging-based evaluation of cardiac function. Serum lactate, a cornerstone of circulatory shock assessment, reflects the net balance of tissue oxygen delivery and consumption, with elevated levels carrying strong prognostic significance across critical illness syndromes [1, 2]. Independently, echocardiographic assessment of right ventricular function has emerged as a powerful predictor of adverse outcomes, with RV dysfunction documented in 20–50% of ICU patients depending on the definition applied and the population studied [3–5].

In routine clinical practice, clinicians encounter a common but under-characterized scenario: patients whose lactate levels and echocardiographic findings appear discordant. A patient with normal lactate but echocardiographic evidence of RV dysfunction presents a fundamentally different clinical challenge from one with elevated lactate and preserved biventricular function. Yet current risk stratification approaches treat these markers independently, potentially missing important hemodynamic phenotypic information embedded in their interaction [6].

Right ventricular function has emerged as a critical determinant of outcomes in critical illness, with RV dysfunction documented across sepsis, ARDS, pulmonary embolism, and post-cardiac surgery populations [4, 7, 8]. However, the prognostic significance of RV dysfunction may vary substantially depending on the overall hemodynamic context—specifically, whether it occurs in the presence or absence of systemic hypoperfusion as reflected by lactate elevation.

We hypothesized a priori that cross-classifying critically ill patients by serum lactate and qualitative RV function would identify clinically distinct groups with differential mortality associations. The four-group framework and the 2.0 mmol/L lactate threshold were specified in the study protocol before the present outcome analyses; the focused collapse of qualitative RV labels into preserved versus abnormal function was an analytic operationalization of that framework. We evaluated whether RV function added prognostic information to lactate and clinical predictors, using MIMIC-IV-ECHO for derivation and an eICU analysis as supportive assessment of the prognostic ordering rather than direct echocardiographic validation.

## Methods

### Study design and data source

This was a retrospective cohort study using the Medical Information Mart for Intensive Care IV (MIMIC-IV) version 2.2 clinical database [9] linked with the MIMIC-IV-ECHO v1.0 echocardiography module [10]. MIMIC-IV contains comprehensive clinical data from patients admitted to Beth Israel Deaconess Medical Center (2008–2019). The MIMIC-IV-ECHO module provides structured echocardiographic measurements from 206,488 transthoracic and transesophageal echocardiographic studies. Supportive external assessment was performed using the eICU Collaborative Research Database v2.0 [11], comprising 200,859 ICU admissions from 208 hospitals across the United States. The study was approved under the PhysioNet credentialing process [12]; individual consent was waived due to the de-identified nature of the data.

### Study population

We included adult patients (≥ 18 years) admitted to an ICU with at least one transthoracic echocardiogram (TTE) performed during the ICU admission and a serum lactate measurement within ± 6 h. Each eligible TTE was paired with its nearest lactate measurement; multiple eligible TTE observations from the same ICU stay and multiple ICU admissions from the same patient were retained. This yielded 6,228 observations from 4,965 patients. Patient-level dependence was addressed with cluster-robust standard errors, and a sensitivity analysis retained only the earliest eligible observation per patient. Exclusion criteria were missing qualitative RV function assessment and age < 18 years.

### Exposure: lactate–RV discordance phenotypes

The primary exposure was a four-category phenotype defined by the cross-classification of:

- *Serum lactate*: Categorized as low (< 2 mmol/L) or elevated (≥ 2 mmol/L), consistent with the prespecified protocol and Surviving Sepsis Campaign thresholds. Continuous log2-transformed lactate was used in the incremental-prediction analysis to avoid discarding prognostic information through dichotomization.
- *RV function*: Categorized from the structured qualitative field in MIMIC-IV-ECHO. Preserved function comprised ‘normal’ and ‘low normal’ labels; all other non-preserved labels in the frozen extraction were assigned to the abnormal-function group [13]. The distribution was: normal 3,338 (53.6%), low normal 387 (6.2%), mild global hypokinesis 713 (11.4%), nonspecific depressed function 405 (6.5%), moderate hypokinesis 624 (10.0%), severe hypokinesis 477 (7.7%), apical free-wall hypokinesis 211 (3.4%), McConnell pattern 49 (0.8%), and hyperdynamic 24 (0.4%). Because hyperdynamic function is not physiologically equivalent to dysfunction, a sensitivity analysis excluded these 24 observations. Qualitative assessments originated from clinical reports in the structured database; source reader credentials, image-level re-adjudication, and inter-reader reliability estimates were unavailable. Among observations with TAPSE, concordance with TAPSE < 1.7 cm was 89.5% (Supplementary Table S3).

The resulting four phenotypes were:

- *Low-Lactate/Preserved-RV*: Normal lactate with normal RV function (reference group).
- *Low-Lactate/RV-Dysfunction*: Normal lactate with echocardiographic evidence of RV dysfunction, potentially compatible with a state of compensated RV dysfunction.
- *High-Lactate/Preserved-RV*: Elevated lactate with preserved RV function, a phenotype pattern consistent with non-RV-mediated mechanisms of hyperlactatemia.
- *High-Lactate/RV-Dysfunction*: Elevated lactate with RV dysfunction, representing concordant hemodynamic compromise.

### Primary outcome

The primary outcome was in-hospital mortality.

### Covariates

#### Demographics

Age and sex.

#### Comorbidities

Congestive heart failure, chronic kidney disease, diabetes mellitus, COPD, liver disease, cancer, and sepsis (ICD-9/10 codes).

#### Treatment intensity

Vasopressor administration within ± 2 h of echocardiography, mechanical ventilation, and CRRT.

#### Laboratory values

Serum creatinine and platelet count nearest to echo time (available in 62.8%).

#### Echo parameters

LVEF (78.4%), LVOT VTI (57.7%), TR gradient (68.0%), TAPSE (19.7%), IVC diameter (23.6%).

### Missing data strategy

Variables with substantial missingness were evaluated in complete-case sensitivity models [14]. The primary models (Models 1 and 2) used variables with near-complete availability (> 99%); Model 3 incorporated laboratory values (62.8% complete), and Model 4 incorporated LVEF (78.4% complete). Because the missingness mechanism could not be established, these analyses may be subject to selection bias. Consistency across models was interpreted as supportive robustness rather than evidence that complete-case analysis is superior to multiple imputation.

### Statistical analysis

Sequential logistic regression assessed associations between phenotype and hospital mortality. Because patients could contribute multiple observations, all primary models used sandwich standard errors clustered by subject_id. A sensitivity analysis retained the chronologically earliest eligible observation for each patient. Model specifications were:

- *Model 1*: Demographics (age, sex) + phenotype.
- *Model 2*: Model 1 + comorbidities (CHF, CKD, sepsis) + treatment intensity (vasopressors, MV, CRRT).
- *Model 3*: Model 2 + laboratory severity (creatinine, platelets; *n* = 3,910).
- *Model 4*: Model 2 + LVEF (*n* = 4,885).

Multiplicative interaction between lactate category and RV function was assessed using a product term in logistic regression. Because hospital mortality was common, additive interaction was quantified from adjusted risk ratios estimated by modified Poisson regression with robust variance: RERI = RR11 − RR10 − RR01 + 1. Its 95% CI used 2,000 patient-level bootstrap resamples. Incremental prognostic value was tested using three nested models. Model A included clinical covariates (age, sex, CHF, CKD, sepsis, vasopressors, mechanical ventilation, and CRRT) and continuous log2-transformed lactate. Model B additionally included binary RV dysfunction. Model C retained continuous log2-transformed lactate and added three indicator variables representing the four-group lactate–RV phenotype, thereby allowing threshold-based and joint lactate–RV information beyond the continuous lactate term. We report ΔAUC and ΔBrier score with 95% CIs from 500 patient-level bootstrap resamples, likelihood-ratio testing, and calibration. Cross-validation folds were grouped by patient. Sensitivity analyses included alternative lactate thresholds, a ± 3-hour pairing window, one observation per patient, exclusion of hyperdynamic RV labels, and adjustment for maximum hourly SOFA during the first 24 ICU hours. Exploratory analyses used the eligible TTE–lactate observation as the index unit unless explicitly described as patient-level. The serial-lactate analysis counted unique patients with at least two measurements from 24 h before through 48 h after the index TTE and summarized change over ± 6 h. Restricted cubic spline models used five knots at the 10th, 25th, 50th, 75th, and 90th percentiles of the lactate distribution; severity trends used ordinal scores and Spearman correlation. Vasopressor-stratified comparisons and decision curves were descriptive. The original exploratory bootstrap validation used 500 resamples; revision analyses resampled patients. All analyses used Python; a two-sided *p* < 0.05 was considered significant.

### Supportive external assessment

The eICU Collaborative Research Database was used for supportive external assessment. We included adult ICU stays with male or female sex recorded, a lactate measurement during the first 24 h, and an observed hospital discharge status; the first qualifying lactate was used. Hospital mortality was defined as hospitaldischargestatus=Expired. RV disease was identified from diagnosis strings for pulmonary hypertension, cor pulmonale, or pulmonary hypertension/RV failure. This diagnosis-coded construct is not equivalent to contemporaneous echo-defined RV dysfunction and under-ascertains subclinical disease. Models used robust standard errors clustered by unique patient identifier. The age-sex model was followed by an aligned clinical model including age, sex, CHF, CKD, sepsis, vasopressor infusion, mechanical ventilation, and dialysis; a sensitivity model additionally assessed APACHE IVa severity among complete cases. Because RV ascertainment and cohort case mix differed, eICU and MIMIC effect estimates were not treated as directly interchangeable.

## Results

### Study population

From 179,928 TTE studies in MIMIC-IV-ECHO, 7,038 eligible TTE–lactate observations were identified. After excluding 810 without qualitative RV assessment, 6,228 observations from 4,965 patients, 5,309 hospital admissions, and 5,434 ICU stays formed the analytic cohort. Overall, 847 patients contributed more than one observation, 388 contributed more than one ICU stay, and 570 ICU stays contained more than one qualifying TTE observation. The cohort had a median age of 66 years (IQR 54–76), 57.8% were male, and hospital mortality was 30.9% (1,927/6,228).

### Distribution of discordance phenotypes

The four phenotypes were distributed: Low-Lactate/Preserved-RV (*n* = 2,221, 35.7%), Low-Lactate/RV-Dysfunction (*n* = 1,258, 20.2%), High-Lactate/Preserved-RV (*n* = 1,504, 24.1%), and High-Lactate/RV-Dysfunction (*n* = 1,245, 20.0%).

### Baseline characteristics

Table 1 presents baseline characteristics. Patients with RV dysfunction had higher CHF prevalence and lower LVEF; patients with elevated lactate had higher sepsis prevalence and greater treatment intensity.

**Table 1:** Baseline characteristics by lactate–RV discordance phenotype

Variable	LowLac/PresRV (*n* = 2221)	LowLac/RVdys (*n* = 1258)	HiLac/PresRV (*n* = 1504)	HiLac/RVdys (*n* = 1245)	*p*
Age, years	66 [54–76]	65 [54–74]	64 [53–75]	67 [56–76]	0.015
Male sex, *n* (%)	1205 (54.3)	808 (64.2)	805 (53.5)	783 (62.9)	< 0.001
Mortality, *n* (%)	418 (18.8)	318 (25.3)	603 (40.1)	588 (47.2)	< 0.001
Vasopressor, *n* (%)	734 (33.0)	596 (47.4)	750 (49.9)	650 (52.2)	< 0.001
MV, *n* (%)	1087 (48.9)	615 (48.9)	847 (56.3)	678 (54.5)	< 0.001
CRRT, *n* (%)	279 (12.6)	224 (17.8)	317 (21.1)	295 (23.7)	< 0.001
Lactate, mmol/L	1.3 [1.0-1.6]	1.3 [1.1–1.6]	2.9 [2.3–4.4]	3.3 [2.4–5.3]	< 0.001
CHF, *n* (%)	871 (39.2)	921 (73.2)	542 (36.0)	837 (67.2)	< 0.001
CKD, *n* (%)	633 (28.5)	501 (39.8)	376 (25.0)	461 (37.0)	< 0.001
Sepsis, *n* (%)	761 (34.3)	408 (32.4)	733 (48.7)	549 (44.1)	< 0.001
LVEF, %	55 [50–65]	40 [25–55]	55 [45–65]	35 [20–55]	< 0.001
LVOT VTI, cm	21 [17–25]	17 [13–21]	20 [16–25]	14 [11–18]	< 0.001
TAPSE, cm	2.1 [1.8–2.4]	1.3 [1.1–1.5]	2.0 [1.8–2.3]	1.3 [1.1–1.5]	< 0.001
TR gradient, mmHg	31 [25–39]	34 [26–46]	30 [23–37]	33 [25–41]	< 0.001
Creatinine, mg/dL	1.1 [0.8-2.0]	1.4 [0.9–2.4]	1.4 [1.0-2.3]	1.7 [1.2–2.7]	< 0.001
Platelets, K/µL	195 [138–277]	185 [129–246]	162 [97–240]	174 [111–242]	< 0.001

Continuous variables: median [IQR]; categorical: *n* (%)

### Mortality by discordance phenotype

**Fig. 1:**
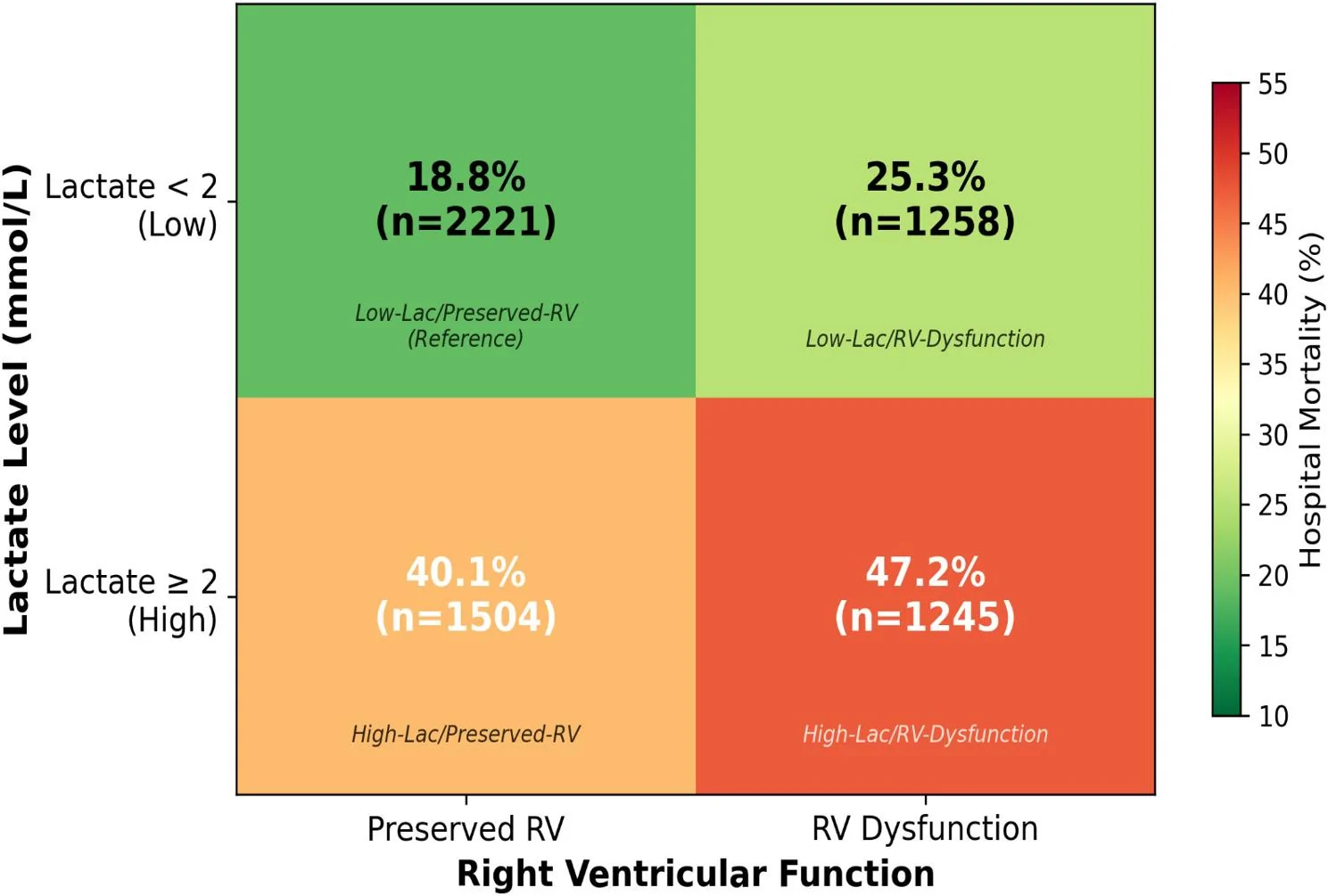
Hospital mortality by lactate–RV group. The heatmap displays descriptive mortality rates across the 2×2 classification; formal interaction estimates and confidence intervals are reported separately

Hospital mortality demonstrated a stepwise gradient (Fig. 1): Low-Lactate/Preserved-RV 18.8%, Low-Lactate/RV-Dysfunction 25.3%, High-Lactate/Preserved-RV 40.1%, and High-Lactate/RV-Dysfunction 47.2% (*p* < 0.001). The absolute mortality difference associated with RV dysfunction was 6.5% points in the low-lactate stratum and 7.1% points in the high-lactate stratum. These descriptive contrasts do not, by themselves, establish additive interaction.

### Multivariable analysis

**Fig. 2:**
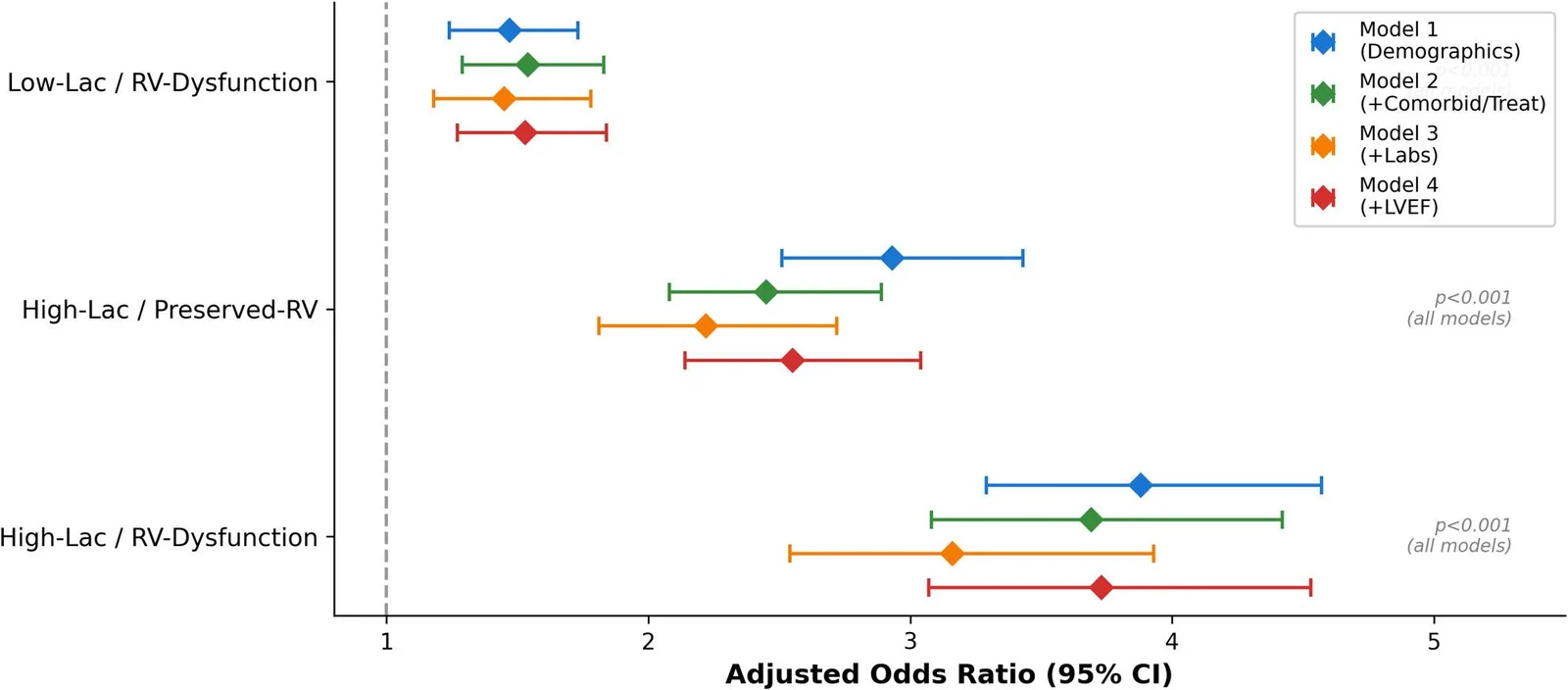
Stability of Phenotype OR Across Sequentially Adjusted Models. (Reference: Low-Lactate / Preserved-RV). Phenotype odds ratios across sequentially adjusted models. Primary inference uses patient-clustered robust standard errors (Supplementary Table S6). Wider intervals in Models 3–4 reflect reduced complete-case sample sizes

Sequential logistic regression with patient-clustered robust standard errors demonstrated stable phenotype associations (Fig. 2; Table 2; Supplementary Table S6 and Figure S4). In Model 1, ORs were 1.47 (95% CI 1.22–1.77) for Low-Lactate/RV-Dysfunction, 2.93 (2.50–3.42) for High-Lactate/Preserved-RV, and 3.88 (3.28–4.59) for High-Lactate/RV-Dysfunction. In Model 2, corresponding ORs were 1.54 (1.27–1.87), 2.45 (2.08–2.89), and 3.69 (3.07–4.43). Model 3 estimates were 1.45 (1.14–1.85), 2.22 (1.81–2.74), and 3.16 (2.51–3.99); Model 4 estimates were 1.53 (1.23–1.89), 2.55 (2.12–3.06), and 3.73 (3.01–4.61). LVEF was not statistically significant in Model 4 (OR per percentage point 1.00, 95% CI 0.99–1.00; *p* = 0.520), but this result should not be interpreted as evidence that LVEF lacks prognostic importance.

**Table 2:** Adjusted odds ratios for hospital mortality

Phenotype	Model 1 (Demog)	Model 2 (+ Comorbid)	Model 3 (+ Labs)	Model 4 (+ LVEF)
*N*	6,228	6,228	3,910	4,885
LowLac/RV-Dysfxn	1.47***	1.54***	1.45**	1.53***
HiLac/Preserved-RV	2.93***	2.45***	2.22***	2.55***
HiLac/RV-Dysfxn	3.88***	3.69***	3.16***	3.73***
Pseudo-R²	0.054	0.127	0.130	0.133

Reference: Low-Lactate/Preserved-RV. Primary confidence intervals and *p* values use patient-clustered robust standard errors (Supplementary Table S6). Pseudo-R² values are modest and reflect association rather than a comprehensive prediction model

*p < 0.05, **p < 0.01, ***p < 0.001

### Physiological coherence of phenotypes

**Fig. 3:**
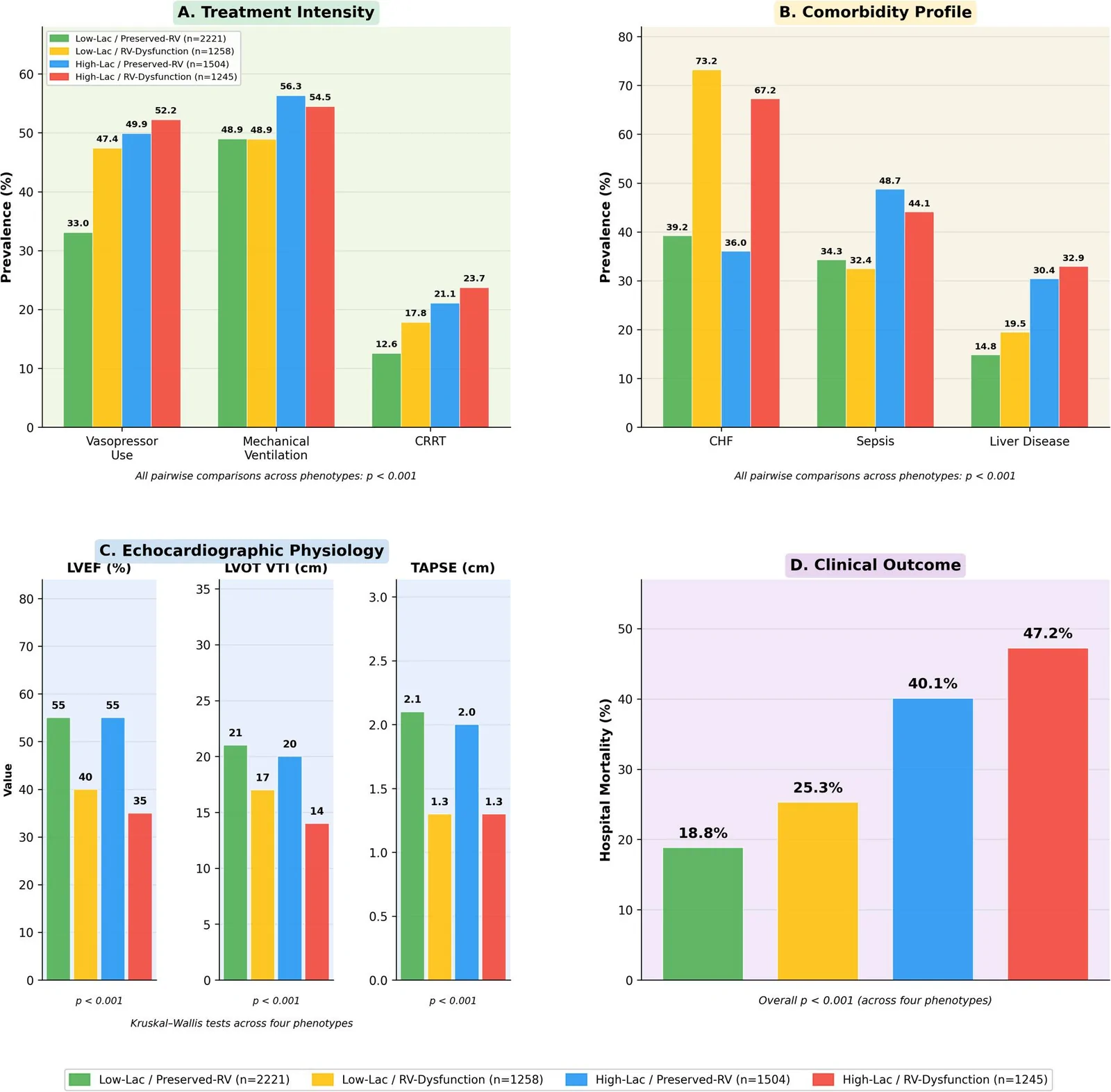
Descriptive Clinical and Physiologic Characterization of Lactate-RV Groups. Panels summarize treatment intensity (**A**), clinical substrate (**B**), echocardiographic physiology (**C**), and mortality (**D**). Comparisons are descriptive and should not be interpreted as independent validation

The phenotypes demonstrated coherent physiological profiles (Fig. 3; Supplementary Figure S8). Low-Lactate/RV-Dysfunction: lower LVEF (40% vs. 55%), reduced VTI (17 vs. 21 cm), highest CHF (73.2%)—potentially compatible with a state of compensated RV dysfunction [7, 8, 15]. High-Lactate/Preserved-RV: highest sepsis (48.7%), lowest platelets (162 K/µL)—a pattern consistent with distributive or impaired lactate-clearance physiology [1, 16]. High-Lactate/RV-Dysfunction: lowest VTI (14 cm), highest creatinine (1.7 mg/dL), highest mortality—may reflect combined metabolic stress and RV dysfunction.

### Treatment heterogeneity

Vasopressor status revealed treatment heterogeneity (Supplementary Figure S5). In Low-Lactate/Preserved-RV, vasopressor use was associated with increased mortality (14.7% without vs. 27.2% with vasopressors), whereas in High-Lactate/RV-Dysfunction, mortality exceeded 55% among patients receiving vasoactive support (37.0% without vs. 56.6% with), which may identify a subgroup with persistent high mortality among patients receiving vasoactive support [17].

Figure 3 descriptively integrates treatment burden, comorbidity substrate, echocardiographic physiology, and clinical outcomes across the four groups; these profiles are exploratory and do not constitute independent phenotype validation.

### Dynamic lactate trajectory

**Fig. 4:**
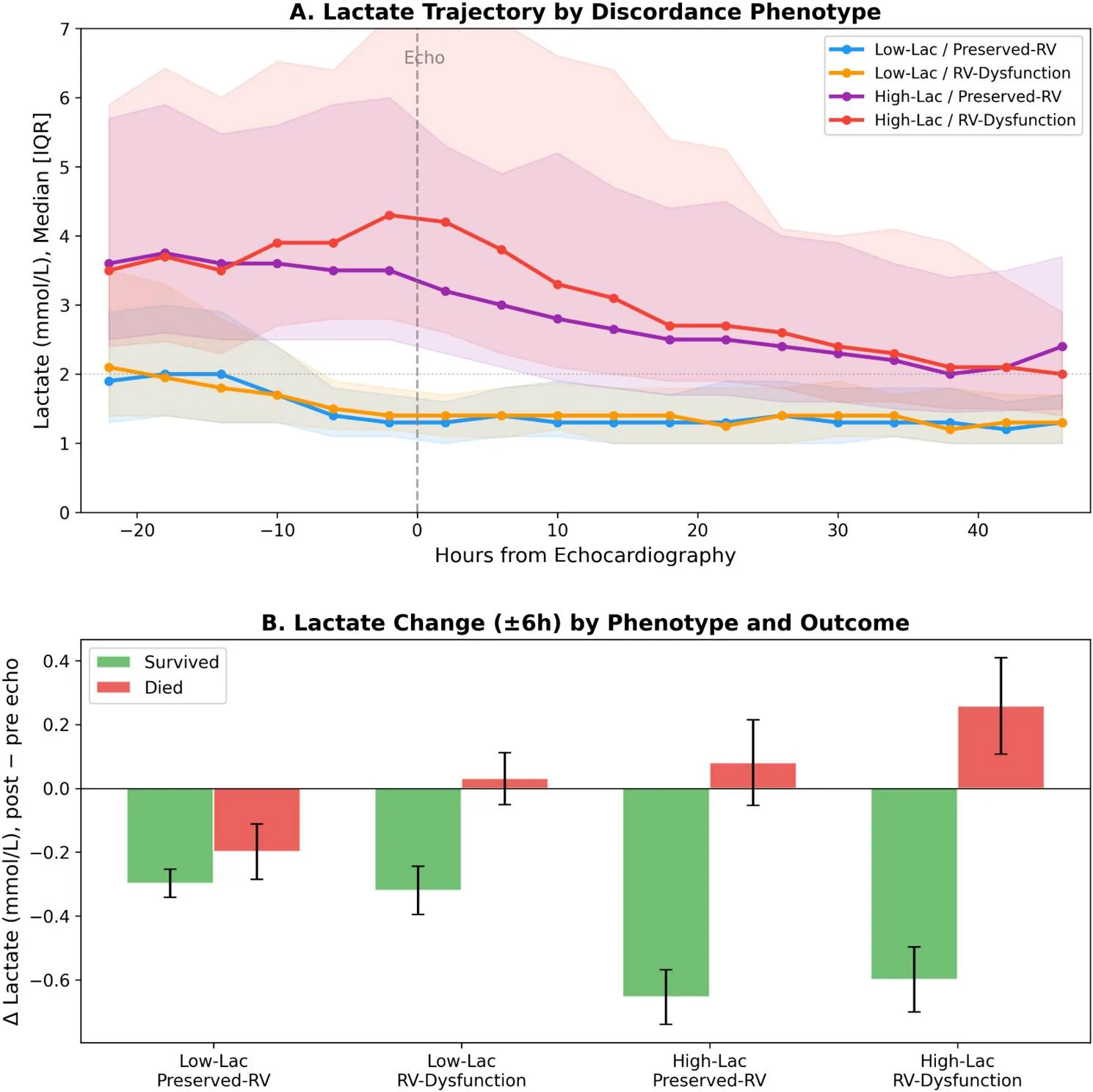
Dynamic Lactate Trajectory by Discordance Phenotype. Panel **A**: Median lactate trajectory (IQR shaded) from 24 hours before to 48 hours after echocardiography, stratified by phenotype. Panel **B**: Post-echo lactate change (±6h) stratified by phenotype and survival outcome. Survivors demonstrate effective lactate clearance while non-survivors show persistent or rising lactate across all high-risk phenotypes

To examine whether the four phenotypes capture distinct physiologic trajectories, we analyzed serial lactate measurements within a − 24 to + 48 h window around the index echocardiogram (*n* = 4,634 patients with ≥ 2 measurements, Fig. 4). The High-Lactate/RV-Dysfunction phenotype demonstrated persistent lactate elevation with minimal clearance (mean Δ − 0.19 mmol/L over ±6 h), whereas the High-Lactate/Preserved-RV phenotype showed partial clearance (Δ − 0.35 mmol/L). The Low-Lactate phenotypes maintained stable low levels throughout the observation period. Among patients who died, lactate rose in the post-echo period across all high-risk phenotypes (High-Lactate/RV-Dysfunction: Δ + 0.26 mmol/L in non-survivors vs. − 0.60 in survivors, *p* < 0.001; High-Lactate/Preserved-RV: Δ + 0.08 vs. − 0.65, *p* < 0.001), consistent with phenotype assignment capturing distinct physiologic trajectories rather than static point-in-time classification.

### Predictive discrimination

Lactate alone was more discriminative than the four-category phenotype (AUC 0.692 vs. 0.648; Supplementary Figure S6), confirming that the phenotype should not be viewed as a replacement for continuous lactate. The revised incremental comparison therefore used the same clinical predictors in both models: clinical covariates plus continuous log2 lactate yielded AUC 0.758 and Brier score 0.1743; adding RV dysfunction yielded AUC 0.761 and Brier score 0.1734. The improvement was statistically detectable but quantitatively small (ΔAUC 0.003, patient-bootstrap 95% CI 0.001–0.006; ΔBrier − 0.0009, 95% CI − 0.0019 to − 0.0003; likelihood-ratio *p* < 0.001). Adding the full four-group phenotype produced a similarly small improvement in discrimination (AUC 0.762; ΔAUC 0.004, 95% CI 0.002–0.007; likelihood-ratio *P* < 0.001), with a 10-fold patient-grouped cross-validated AUC of 0.759 (Supplementary Table S8). In 10-fold cross-validation grouped by patient, AUCs were 0.755, 0.758, and 0.759, Brier scores were 0.1752, 0.1743, and 0.1743, calibration-in-the-large values were − 0.0005, − 0.0003, and − 0.0002, and calibration slopes were approximately 0.98 for Models A, B, and C, respectively (Supplementary Figure S9). Detailed results are in Supplementary Table S8. Additional exploratory spline, calibration, and bootstrap analyses are presented in Supplementary Figures S1–S3; the original exploratory prediction models in Figures S2–S3 are retained as supporting analyses and should not be compared directly with the revised nested models.

### RV severity continuum

The qualitative RV distribution showed a severity gradient: mortality was 27.0% with normal function, 31.7% with mild global hypokinesis, 35.1% with moderate hypokinesis, and 45.7% with severe hypokinesis (p-trend < 0.001). The full distribution, including low-normal and non-ordinal labels, is reported in Supplementary Table S5. Across six qualitative RV categories and five lactate strata, mortality ranged from 10.9% (normal RV, lactate < 1.0 mmol/L) to 90.0% (severe dysfunction, lactate ≥ 8.0 mmol/L) (Supplementary Figure S7), supporting a continuous bivariate severity surface while avoiding over-interpretation of sparse cells.

### Interaction analysis

In the patient-clustered adjusted logistic model, the multiplicative interaction was not significant (interaction OR 0.98, 95% CI 0.76–1.25; *p* = 0.854). From the modified Poisson model, adjusted RR10, RR01, and RR11 were 1.809, 1.357, and 2.237; RERI was 0.070 (2,000-resample patient-bootstrap 95% CI − 0.177 to 0.284). The interval included zero, providing no clear evidence of additive interaction. Detailed interaction estimates are provided in Supplementary Table S4.

### Sensitivity analyses

Results were directionally consistent across sensitivity analyses. Alternative lactate thresholds and the ± 3-hour pairing window preserved the ordering (Supplementary Tables S1–S2). When restricted to the earliest eligible observation per patient (*n* = 4,965), adjusted ORs were 1.45 (95% CI 1.18–1.79), 2.41 (2.03–2.87), and 3.77 (3.12–4.55). After additional adjustment for maximum hourly SOFA during the first ICU day (*n* = 6,052 observations; 4,810 patients), corresponding ORs were 1.51 (1.24–1.83), 2.28 (1.93–2.69), and 3.27 (2.71–3.95). Excluding 24 hyperdynamic observations yielded ORs of 1.54 (1.27–1.86), 2.45 (2.08–2.89), and 3.68 (3.06–4.43). The one-observation-per-patient, SOFA-adjusted, and hyperdynamic-exclusion analyses are presented in Supplementary Tables S7, S10, and S11, respectively.

### Supportive external assessment

**Fig. 5:**
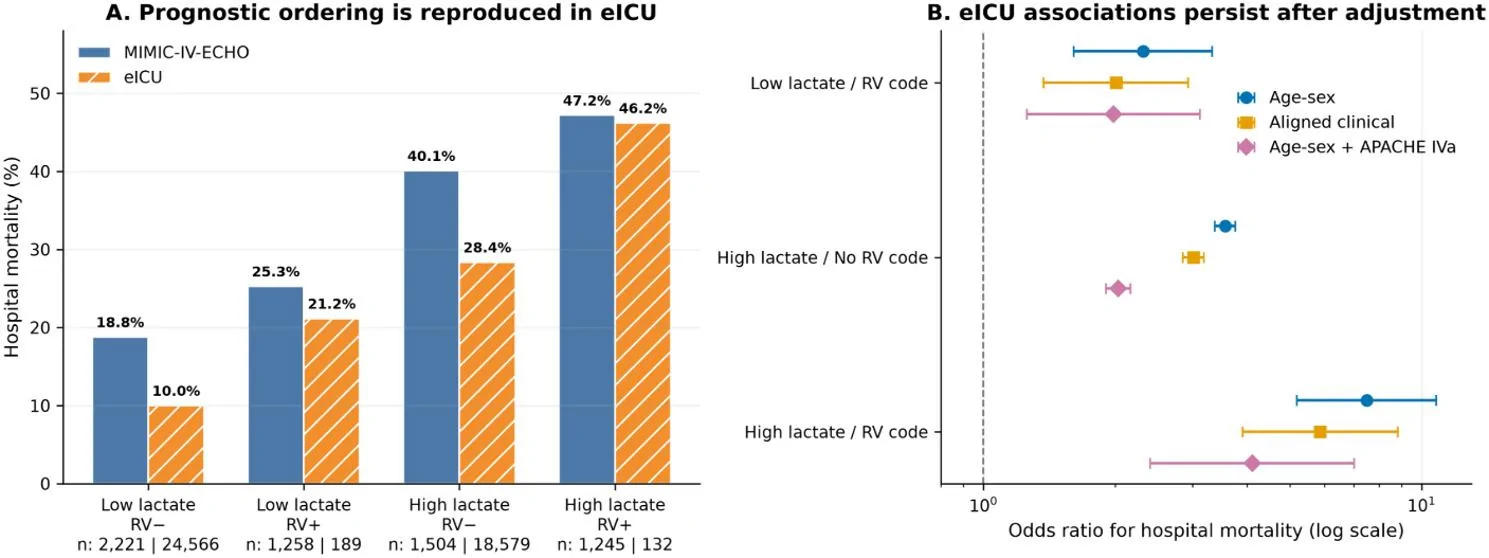
Supportive External Assessment in eICU. Panel **A** shows hospital mortality ordering in MIMIC-IV-ECHO and eICU. Panel **B** shows eICU-only patient-clustered ORs from age-sex, aligned clinical, and APACHE IVa models. Diagnosis-coded eICU RV disease is not equivalent to echo-defined RV dysfunction. Panel **A** sample sizes are shown as n: MIMIC-IV-ECHO | eICU; RV-/RV+ are echo-defined in MIMIC-IV-ECHO and diagnosis-code-defined in elCU. Panel **B** reference is Low lactate/RV code- (OR=1; omitted). Aligned model: age, sex, CHF, CKD, sepsis, vasopressors, ventilation, and dialysis

The reconstructed eICU cohort included 43,466 ICU stays from 38,200 patients and 194 hospitals after excluding 380 stays without observed hospital discharge status. Diagnosis-coded RV disease was present in 321 stays (0.74%). Hospital mortality followed the same ordering (Table 3; Fig. 5): 10.0% (Low-Lactate/No-RV-code), 21.2% (Low-Lactate/RV-code), 28.4% (High-Lactate/No-RV-code), and 46.2% (High-Lactate/RV-code). With patient-clustered age-sex adjustment, ORs were 2.31 (95% CI 1.61–3.33), 3.56 (3.37–3.76), and 7.49 (5.19–10.80). In the aligned clinical model, ORs were 2.01 (1.37–2.93), 3.01 (2.85–3.19), and 5.87 (3.91–8.82). An APACHE IVa sensitivity model (*n* = 37,047) preserved the ordering: 1.98 (1.26–3.12), 2.03 (1.90–2.16), and 4.10 (2.40–7.02). These results support replication of the prognostic pattern, not direct validation of the echocardiographic phenotype. Complete external-assessment results are provided in Supplementary Table S9.

**Table 3:** Supportive external assessment in eICU (*n* = 43,466; 38,200 patients; 194 hospitals)

eICU group	*N*	Mortality	Age-sex OR	Aligned OR	APACHE IVa OR
LowLac/No RV code	24,566	10.0%	ref	ref	ref
LowLac/RV code	189	21.2%	2.31 (1.61–3.33)	2.01 (1.37–2.93)	1.98 (1.26–3.12)
HiLac/No RV code	18,579	28.4%	3.56 (3.37–3.76)	3.01 (2.85–3.19)	2.03 (1.90–2.16)
HiLac/RV code	132	46.2%	7.49 (5.19–10.80)	5.87 (3.91–8.82)	4.10 (2.40–7.02)

All ORs use patient-clustered robust standard errors. The aligned model includes age, sex, CHF, CKD, sepsis, vasopressors, ventilation, and dialysis. The APACHE IVa model includes 37,047 complete cases

Absolute mortality differed between cohorts, particularly among patients without an RV code, likely reflecting case mix, selection for echocardiography in MIMIC, inclusion timing, and clinical practice. The eICU RV-code prevalence was only 0.74% versus 40.2% echo-defined dysfunction in MIMIC, confirming marked under-ascertainment. Adjustment sets were aligned as far as available variables permitted, and APACHE IVa sensitivity analysis further addressed illness severity; nevertheless, the different RV constructs preclude direct numerical equivalence. The external analysis is therefore supportive evidence for prognostic ordering.

## Discussion

In this retrospective study of ICU patients selected for formal echocardiography, lactate–RV cross-classification identified four groups with graded mortality. Patient-clustered analyses and a one-observation-per-patient sensitivity analysis produced consistent associations. RV function added statistically detectable but quantitatively modest discrimination beyond continuous lactate and clinical predictors. The eICU analysis reproduced the ordering using diagnosis-coded RV disease, but it does not directly validate the echo-defined phenotype.

Perhaps the most clinically important finding is the Low-Lactate/RV-Dysfunction phenotype (20% of cohort, 25.3% mortality, OR 1.53), which may represent a clinically under-recognized state potentially compatible with a state of compensated RV dysfunction [7, 8]. The phenotype is compatible with preserved systemic compensation despite impaired RV mechanics, where maintained forward flow and hepatic lactate clearance mask underlying hemodynamic vulnerability. The elevated mortality raises the possibility that these patients represent a clinically vulnerable subgroup whose risk is not apparent from lactate values alone.

### Proposed physiologic interpretation

The discordance between lactate and RV function can be understood through the lens of compensatory reserve and the heterogeneity of shock physiology [1, 16, 18, 19]. In the Low-Lactate/RV-Dysfunction phenotype, preserved lactate clearance despite RV impairment is compatible with several non-exclusive mechanisms: (1) maintained LV compensation preserving systemic cardiac output; (2) intact hepatosplanchnic lactate clearance; (3) early-stage RV dysfunction preceding overt forward flow limitation; and (4) chronic neurohormonal adaptation. In the High-Lactate/Preserved-RV phenotype, lactate elevation is consistent with extra-cardiac mechanisms including sepsis-related mitochondrial dysfunction, hepatosplanchnic hypoperfusion, and impaired hepatic clearance [1, 2]. The concordant High-Lactate/RV-Dysfunction phenotype is consistent with exhaustion of compensatory reserves, positioning the four phenotypes along a physiologic continuum of progressive hemodynamic compromise.

Neither interaction scale provided clear evidence of departure from additivity or multiplicativity. The multiplicative interaction was not significant, and the RR-based RERI was 0.070 (patient-bootstrap 95% CI − 0.177 to 0.284). These results do not support claims of biological synergy or additive risk amplification.

The dynamic lactate trajectory analysis suggests that the groups capture differing physiologic courses. For prediction, however, continuous lactate remained stronger than the four-category classification alone. Adding RV dysfunction to a clinical model already containing continuous lactate increased AUC by only 0.003; although its bootstrap CI excluded zero and Brier score improved slightly, the incremental discrimination was limited. Thus, the principal contribution is physiologic stratification rather than material improvement in prediction.

LVEF was not statistically significant after adjustment in the complete-case Model 4, but this null result is imprecise evidence rather than proof of no prognostic importance. LVEF was missing in 21.6%, was substantially lower in RV-dysfunction groups, and may share prognostic information with RV status and other covariates. Collinearity, selection into the complete-case subset, and limited representation of time-varying ventricular function may all attenuate its coefficient.

Prior sepsis and cardiovascular phenotyping studies have identified heterogeneous hemodynamic clusters using multi-variable or data-driven methods [18, 19]. Our narrower contribution is a simple, prespecified, bedside-interpretable cross-classification of a biochemical perfusion marker with qualitative RV assessment. It is not proposed as a replacement for richer clustering approaches or established severity scores.

### Limitations

Several limitations warrant discussion. First, receipt of formal TTE is a strong selection mechanism: patients undergoing echocardiography differ from general ICU populations in suspected cardiovascular disease, hemodynamic instability, and treatment intensity. The results therefore apply to TTE-selected ICU patients and should not be generalized to all ICU admissions. Second, qualitative RV labels were abstracted from routine clinical reports; source reader credentials, image-level re-adjudication, and inter-reader reliability were unavailable. Rare and non-ordinal labels complicate binary mapping, although excluding hyperdynamic observations did not change estimates. Third, repeated observations were handled with patient-clustered inference and a one-observation-per-patient sensitivity analysis, but time-varying confounding remains possible. Fourth, LVEF and laboratory covariates had substantial missingness, and the LVEF null result may reflect missingness, overlap with RV dysfunction, and complete-case selection. Fifth, first-day SOFA adjustment attenuated but did not eliminate associations; incomplete SOFA linkage and residual or time-varying confounding remain. Sixth, retrospective associations do not support causal or treatment-effect claims. Seventh, eICU diagnosis-coded RV disease is not equivalent to echo-defined dysfunction and markedly under-ascertained RV abnormalities (0.74% vs. 40.2%). Although adjustment sets were aligned and APACHE sensitivity analysis was added, the external findings remain supportive replication of ordering rather than direct phenotype validation. Finally, pseudo-R² values of 0.054–0.133 are modest, underscoring that these models are not comprehensive mortality-prediction tools.

## Conclusions

Among ICU patients selected for echocardiography, lactate–RV cross-classification identified four groups with graded mortality associations that were robust to patient clustering, first-day SOFA adjustment, and a single-observation sensitivity analysis. RV dysfunction added only a small increment in discrimination beyond continuous lactate and clinical predictors. RR-based RERI did not show clear additive interaction. Diagnosis-coded eICU findings reproduced the prognostic ordering but did not directly validate the echocardiographic phenotype. The framework is intended for physiologic stratification, not causal inference or standalone prediction.

## Supplementary Information

Supplementary Material 1.

Supplementary Material 2.

## Data Availability

The MIMIC-IV and MIMIC-IV-ECHO datasets are publicly available through PhysioNet (https://physionet.org) after completion of the required credentialing process. The eICU Collaborative Research Database is available through the same platform. The revision analysis code and machine-readable result tables are provided as supplementary files.
