# Supplementary material for: Lactate–right ventricular discordance patterns and mortality in critical illness
Source: BMC Cardiovasc Disord. 2026 Aug 12;26:1047. doi: 10.1186/s12872-026-06440-1 (PMC13746518; doi:10.1186/s12872-026-06440-1)
Supplement: Supplementary file 2 [file 12872_2026_6440_MOESM2_ESM.docx]

**Supplementary Materials**

Lactate–Right Ventricular Discordance Patterns and Mortality in Critical Illness

# Supplementary Tables

## Table S1. Sensitivity Analysis: Alternative Lactate Thresholds

Adjusted odds ratios (Model 2: demographics, comorbidities, treatment intensity) for hospital mortality across three lactate thresholds (1.5, 2.0, and 2.5 mmol/L). The monotonic mortality gradient and phenotype OR ordering are preserved across all thresholds.

| **Phenotype** | **n** | **Mortality** | **OR (95% CI)** |
| --- | --- | --- | --- |
| **Threshold: 1.5 mmol/L** |  |  |  |
| LL/PRV (ref) | 1,473 | 16.4% | 1.00 (reference) |
| LL/RVD | 780 | 22.1% | 1.47 (1.15–1.88) |
| HL/PRV | 2,252 | 34.6% | 2.35 (1.96–2.81) |
| HL/RVD | 1,723 | 42.6% | 3.67 (3.01–4.47) |
| **Threshold: 2.0 mmol/L** |  |  |  |
| LL/PRV (ref) | 2,221 | 18.8% | 1.00 (reference) |
| LL/RVD | 1,258 | 25.3% | 1.54 (1.27–1.87) |
| HL/PRV | 1,504 | 40.1% | 2.45 (2.08–2.89) |
| HL/RVD | 1,245 | 47.2% | 3.69 (3.07–4.43) |
| **Threshold: 2.5 mmol/L** |  |  |  |
| LL/PRV (ref) | 2,716 | 20.7% | 1.00 (reference) |
| LL/RVD | 1,595 | 26.7% | 1.50 (1.27–1.78) |
| HL/PRV | 1,009 | 45.4% | 2.71 (2.28–3.23) |
| HL/RVD | 908 | 52.9% | 4.01 (3.31–4.85) |

All ORs p<0.01. Model adjusted for age, sex, CHF, CKD, sepsis, vasopressors, MV, and CRRT. Standard errors were clustered by subject_id.

## Table S2. Sensitivity Analysis: Stricter Pairing Window (±3 Hours)

Restricting the echo–lactate pairing window from ±6 hours to ±3 hours yielded 4,398 observations from 3,614 patients in the final frozen cohort. Patient-clustered estimates preserved the phenotype ordering.

| **Phenotype** | **n** | **Mortality** | **OR (95% CI)** | **p** |
| --- | --- | --- | --- | --- |
| LL/PRV (ref) | 1,348 | 20.8% | 1.00 (reference) | — |
| LL/RVD | 851 | 27.0% | 1.59 (1.26–2.00) | <0.001 |
| HL/PRV | 1,174 | 43.1% | 2.53 (2.09–3.07) | <0.001 |
| HL/RVD | 1,025 | 48.8% | 3.58 (2.90–4.41) | <0.001 |

Model 2 adjusted for age, sex, CHF, CKD, sepsis, vasopressors, MV, and CRRT. Standard errors were clustered by subject_id.

## Table S3. Concordance Between Qualitative RV Assessment and TAPSE

Among 1,226 observations from 1,113 patients with both qualitative RV function assessment and TAPSE measurement, concordance between qualitative RV dysfunction and TAPSE <1.7 cm was 89.5%.

|  | **TAPSE ≥1.7 cm** | **TAPSE <1.7 cm** | **Total** |
| --- | --- | --- | --- |
| Qualitative: Preserved | 630 | 73 | 703 |
| Qualitative: Dysfunction | 56 | 467 | 523 |
| **Total** | **686** | **540** | **1,226** |

*Concordance: 89.5%. Sensitivity: 89.3%. Specificity: 89.6%. PPV: 86.5%. NPV: 91.8%.*

TAPSE <1.7 cm threshold based on ASE/EACVI guidelines (Rudski et al., JASE 2010). Concordance provides partial convergent evidence but does not replace image-level adjudication or reader-reliability assessment.

## Table S4. Interaction Analysis Between Lactate and RV Dysfunction

Formal assessment of interaction between elevated lactate (≥2 mmol/L) and RV dysfunction on hospital mortality. Multiplicative interaction used patient-clustered logistic regression. Additive interaction used adjusted risk ratios from modified Poisson regression; the RERI 95% CI used 2,000 patient-level bootstrap resamples.

| **Variable** | **Model** | **OR (95% CI)** | **p** |
| --- | --- | --- | --- |
| Lactate ≥2 mmol/L | Main effects | 2.45 (2.08–2.89) | <0.001 |
| RV dysfunction | Main effects | 1.54 (1.27–1.87) | <0.001 |
| Lactate × RV (interaction) | Interaction | 0.98 (0.76–1.25) | 0.854 |
| LR test (1 df) |  | χ² = 0.037 | 0.848 |
| RERI | Additive scale | 0.070 (95% CI −0.177 to 0.284) | Modified Poisson; patient bootstrap |

Models adjusted for age, sex, CHF, CKD, sepsis, vasopressors, MV, and CRRT. RERI = RR11 − RR10 − RR01 + 1; CI resampling unit was subject_id.

## Table S5. RV Severity Dose-Response: Mortality by Qualitative RV Function Category

Hospital mortality across qualitative RV function categories. Ordered hypokinesis categories demonstrate a dose-response pattern; low-normal and rare non-ordinal labels are shown separately to characterize the source field transparently.

| RV Function Category | n (%) | Mortality | Analysis role |
| --- | --- | --- | --- |
| Normal RV function | 3,338 (53.6%) | 27.0% | Reference |
| Low normal function | 387 (6.2%) | 30.7% | Preserved mapping |
| Mild global RV hypokinesis | 713 (11.4%) | 31.7% | Ordered |
| RV function depressed | 405 (6.5%) | 34.8% | Non-specific |
| Moderate global RV hypokinesis | 624 (10.0%) | 35.1% | Ordered |
| Severe global hypokinesis | 477 (7.7%) | 45.7% | Ordered |
| Apical free-wall hypokinesis | 211 (3.4%) | 36.5% | Non-ordinal |
| McConnell pattern | 49 (0.8%) | 28.6% | Non-ordinal |
| Hyperdynamic | 24 (0.4%) | 45.8% | Rare/non-ordinal |

The primary ordered comparison was normal, mild, moderate, and severe global hypokinesis (p-trend<0.001). Nonspecific depressed function and rare patterns (apical free-wall hypokinesis, McConnell pattern, and hyperdynamic) were not forced into the ordinal trend.

# Table S6. Sequential Models With Patient-Clustered Robust Standard Errors

Odds ratios (95% CIs) for the three non-reference groups. Reference: Low-Lactate/Preserved-RV.

| Model | n | LowLac/RVdys | HiLac/PresRV | HiLac/RVdys |
| --- | --- | --- | --- | --- |
| Model 1 | 6,228 | 1.47 (1.22–1.77) | 2.93 (2.50–3.42) | 3.88 (3.28–4.59) |
| Model 2 | 6,228 | 1.54 (1.27–1.87) | 2.45 (2.08–2.89) | 3.69 (3.07–4.43) |
| Model 3 | 3,910 | 1.45 (1.14–1.85) | 2.22 (1.81–2.74) | 3.16 (2.51–3.99) |
| Model 4 | 4,885 | 1.53 (1.23–1.89) | 2.55 (2.12–3.06) | 3.73 (3.01–4.61) |

Models follow the main-text sequential adjustment sets. Standard errors are clustered by subject_id.

# Table S7. Sensitivity Analysis Restricted to One Observation per Patient

The chronologically earliest eligible observation was retained for each of 4,965 patients. Model 2 covariates were used.

| Group | OR (95% CI) | p |
| --- | --- | --- |
| Low-Lactate/Preserved-RV | 1.00 (reference) | — |
| Low-Lactate/RV-Dysfunction | 1.45 (1.18–1.79) | <0.001 |
| High-Lactate/Preserved-RV | 2.41 (2.03–2.87) | <0.001 |
| High-Lactate/RV-Dysfunction | 3.77 (3.12–4.55) | <0.001 |

# Table S8. Incremental Prognostic Value Beyond Continuous Lactate

The three models use the same 6,228 observations and clinical predictors. Confidence intervals for changes use 500 patient-level bootstrap resamples; cross-validation folds are grouped by patient.

| Metric | Model A: Clinical + log2 lactate | Model B: + RV dysfunction | Model C: + four-group phenotype |
| --- | --- | --- | --- |
| AUC | 0.758 | 0.761 | 0.762 |
| Brier score | 0.1743 | 0.1734 | 0.1732 |
| 10-fold grouped-CV AUC | 0.755 | 0.758 | 0.759 |
| 10-fold grouped-CV Brier | 0.1752 | 0.1743 | 0.1743 |
| CV calibration-in-the-large | −0.0005 | −0.0003 | −0.0002 |
| CV calibration slope | ≈0.98 | ≈0.98 | ≈0.98 |
| Likelihood-ratio test | — | χ²=24.88; p<0.001 | χ²=29.54; p<0.001 |

*ΔAUC versus Model A was +0.003 (95% CI 0.001–0.006) for Model B and +0.004 (0.002–0.007) for Model C, from 500 patient-level bootstrap resamples; the likelihood-ratio test was χ²=24.9 (df=1) for Model B and χ²=29.5 (df=3) for Model C, both P<0.001. Calibration slopes are reported to two decimals (≈0.98).*

Clinical predictors: age, sex, CHF, CKD, sepsis, vasopressors, mechanical ventilation, and CRRT.

# Table S9. Reconstructed eICU Supportive External Assessment

Hospital mortality was defined as hospitaldischargestatus=Expired. Models use robust standard errors clustered by unique patient identifier. Diagnosis-coded RV disease is not equivalent to echo-defined RV dysfunction.

| eICU group | N | Mortality | Age-sex OR | Aligned OR | APACHE IVa OR |
| --- | --- | --- | --- | --- | --- |
| LowLac/No RV code | 24,566 | 10.0% | ref | ref | ref |
| LowLac/RV code | 189 | 21.2% | 2.31 (1.61–3.33) | 2.01 (1.37–2.93) | 1.98 (1.26–3.12) |
| HiLac/No RV code | 18,579 | 28.4% | 3.56 (3.37–3.76) | 3.01 (2.85–3.19) | 2.03 (1.90–2.16) |
| HiLac/RV code | 132 | 46.2% | 7.49 (5.19–10.80) | 5.87 (3.91–8.82) | 4.10 (2.40–7.02) |

Aligned model: age, sex, CHF, CKD, sepsis, vasopressor infusion, mechanical ventilation, and dialysis (n=43,466). APACHE model: age, sex, and APACHE IVa score (n=37,047).

# Table S10. MIMIC First-Day SOFA Sensitivity Analysis

The Model 2 adjustment set was augmented with maximum hourly SOFA during the first 24 ICU hours. The locally derived hourly SOFA table linked 6,052 observations from 4,810 patients; standard errors were clustered by patient.

| Group | OR (95% CI) | p |
| --- | --- | --- |
| Low-Lactate/Preserved-RV | 1.00 (reference) | — |
| Low-Lactate/RV-Dysfunction | 1.51 (1.24–1.83) | <0.001 |
| High-Lactate/Preserved-RV | 2.28 (1.93–2.69) | <0.001 |
| High-Lactate/RV-Dysfunction | 3.27 (2.71–3.95) | <0.001 |

SOFA was independently associated with mortality (OR 1.08 per point, 95% CI 1.05–1.11).

# Table S11. Sensitivity Analysis Excluding Hyperdynamic RV Function

The 24 observations labeled hyperdynamic were excluded because hyperdynamic function is not physiologically synonymous with dysfunction. Model 2 covariates and patient-clustered robust standard errors were used (n=6,204).

| Group | OR (95% CI) | p |
| --- | --- | --- |
| Low-Lactate/Preserved-RV | 1.00 (reference) | — |
| Low-Lactate/RV-Dysfunction | 1.54 (1.27–1.86) | <0.001 |
| High-Lactate/Preserved-RV | 2.45 (2.08–2.89) | <0.001 |
| High-Lactate/RV-Dysfunction | 3.68 (3.06–4.43) | <0.001 |

Analysis-unit note for Figures S1–S8: unless a legend states otherwise, the index unit is an eligible TTE–lactate observation from the frozen cohort; repeated observations from the same patient were retained, and figures were not restricted to each patient's first observation. The serial-lactate trajectory analysis is the exception and reports unique patients with at least two measurements around the index TTE. Descriptive panels do not provide independent-patient inference. Revised regression inference clustered standard errors by subject_id, and revision bootstrap and cross-validation procedures operated at the patient level.

# Supplementary Figures

## Figure S1. Restricted Cubic Spline Analysis

Figure S1. Restricted cubic spline analysis of lactate–mortality relationships stratified by RV function. Panel A shows non-linear curves with 95% confidence bands. Panel B shows the descriptive model-based difference in predicted mortality between RV groups across lactate values; it should not be interpreted as a causal attributable effect. The multiplicative interaction test was non-significant.

## Figure S2. Model Calibration and Decision Curve Analysis

Figure S2. Calibration and decision-curve results from the original exploratory baseline and phenotype models. These models are not the nested clinical-plus-continuous-lactate models used for the revised incremental-value analysis and are retained only as exploratory supporting material. The primary nested comparison and patient-grouped cross-validation are reported in Supplementary Table S8.

## Figure S3. Bootstrap Internal Validation

Figure S3. Bootstrap internal validation of the original exploratory baseline-plus-phenotype model (500 resamples). This is not the nested model used for the revised incremental-value analysis; corresponding metrics should not be compared directly with Supplementary Table S8. The figure is retained as exploratory supporting material.

## Figure S4. Forest Plot of Adjusted Odds Ratios (Model 2)

**Figure S4.** Forest plot of adjusted odds ratios (Model 2). All three non-reference phenotypes demonstrate significant mortality associations.

## Figure S5. Hospital Mortality Stratified by Vasopressor Status

Figure S5. Hospital mortality stratified by vasopressor status. Descriptive mortality differences across phenotypes are shown without treatment-effect or causal interpretation.

## Figure S6. Exploratory ROC Comparison

Figure S6. Exploratory ROC comparison. Lactate alone outperformed the four-category phenotype alone. The revised nested comparison, reported in Supplementary Table S8, evaluates whether RV dysfunction adds value to a clinical model already containing continuous lactate; the improvement is statistically detectable but small.

## Figure S7. Hospital Mortality by RV Severity × Lactate Level

**Figure S7.** Hospital Mortality by RV Severity × Lactate Level. Heatmap of mortality rates across six qualitative RV function categories and five lactate strata. The monotonic gradient (Spearman r=0.239, p<10⁻⁶²) supports the biological plausibility of the 2×2 phenotype classification.

## Figure S8. Echocardiographic Parameters by Discordance Phenotype

**Figure S8.** Echocardiographic Parameters by Discordance Phenotype. Boxplots of LVEF, LVOT VTI, TR gradient, and TAPSE across the four phenotype groups. Patients with RV dysfunction demonstrate lower LVEF, reduced VTI, and lower TAPSE regardless of lactate category, while TR gradient shows more modest variation. These individual parameter distributions complement the integrated validation presented in Figure 3.

**Figure S9.** Calibration of the incremental prediction models. Observed in-hospital mortality was plotted against mean predicted mortality within deciles of 10-fold patient-grouped cross-validated predictions. Model A included clinical covariates and continuous log2-transformed lactate; Model B additionally included binary RV dysfunction; and Model C additionally included the full four-group lactate–RV phenotype. The dashed line represents perfect calibration.
